# Human–Robot Collaboration in Industrial Automation: Sensors and Algorithms

**DOI:** 10.3390/s22155848

**Published:** 2022-08-05

**Authors:** Anne Schmitz

**Affiliations:** Engineering and Technology Department, University of Wisconsin-Stout, Menomonie, WI 54751, USA; schmitzann@uwstout.edu

Technology is changing the manufacturing world. For example, sensors are being used to track inventory from the manufacturing floor to a retail shelf or a customer’s door, i.e., asset tracking [1]. These types of interconnected systems constitute the so-called fourth industrial revolution, i.e., Industry 4.0, and are projected to lower manufacturing costs [2]. As the manufacturing industry moves toward these integrated technologies and lower costs, engineers will need to connect these systems via the Internet of Things (IoT) [2]. These engineers will also need to design connected systems that can efficiently and safely interact with humans during the manufacturing process, e.g., a car assembly line [3]. The focal points of this Special Issue are the smart sensors that enable robots and humans to “see” each other [4,5,6,7,8,9] and the machine learning algorithms that process these complex data so the robot can make decisions [10,11,12,13].

One of the biggest challenges in human–robot collaborations is the unpredictability of human actions [14]. To address this challenge, sensors have been integrated into this collaboration to allow the robot and human operator to “see” each other. The most common way for robots to “see” humans is through three-dimensional cameras, e.g., Microsoft Kinect [15]. These data are then used to help the robots detect humans and avoid collisions. In this Special Issue, Khawaja demonstrates the use of this technology to predict human motion [5]. Based on this predicted path, the robot can follow the operator’s movements and be prepared to quickly execute the next step in the task, e.g., tightening a bolt or attaching grommets. This motion prediction framework has been shown to decrease cycle time by up to 25% in the sample task studied (delivering parts and tools to a worker in an automobile assembly task). Another way for the robot to “see” the operator is through a two-dimensional camera. These cameras tend to be used in applications where robots and humans coexist. Yamakawa extended the use of two-dimensional cameras to collaborative applications [4]. A high-speed camera can be used to take images of the operator’s hands, which are then processed quickly and accurately using machine learning. This imaging process has been shown to estimate the operator’s grasp type in 0.07 milliseconds with 94% accuracy. One limitation of using red–green–blue (RGB) imaging is the difficulty in distinguishing between humans in the foreground and moving objects in the background. Himmelsbach addressed this limitation in the field using thermal imaging [6]. This is especially advantageous for situations where robots can “see” both the operator’s workspace and walkways with roaming autonomous vehicles. These autonomous vehicles may inadvertently trigger the robot to slow down or stop. Incorporating thermal imaging allows robots to ignore these roaming robots in the background, resulting in a 50% increase in efficiency. Typically, only a single sensing modality is used to enable the robot to “see” the human operator because these data are difficult to process in real time [14,15]. Amin combined both visual and tactile sensors with the aid of machine learning to quickly process these robust data [8]. Multiple Microsoft Kinect cameras were used to detect a whole human body, while multiple cameras allowed for monitoring a larger workspace. The data from these cameras were fed into a neural network model to determine whether the operator was passing through the workspace, observing the robot, moving too close to the robot for it to work, or interacting with the robot. Tactile sensors on the robot provided additional information about the operator: no interaction, intentional contact, or incidental contact. These systems combined were able to “see” and “feel” the operator with 99% accuracy. Besides human-to-robot communication, messaging the other way from robot to human is also important because humans can become nervous around fast-moving robots. To address this, Grushko studied how robots can use haptic feedback to “talk” to a human [7]. Operators showed a 45% improvement in completion time when haptic feedback was used to inform the operator of the robot’s planned trajectory. The feedback was provided through vibrations on a wearable device on the operator’s glove. Another way humans and robots interact is through teaching, e.g., when the operator teaches the robot to perform a task. Typically, a robot is taught to perform a non-contact task such as spraying. Tasks that involve contact, such as picking up an object, require synchronous sensing or both traction and contact. Zhang developed a sensor that measures both of these forces using a single sensor, as opposed to a multiple-sensor arrangement [9]. This compact sensor arrangement utilizes strain gauges mounted on a cylindrical sleeve. This sensor was validated for a drawer-opening experiment where the robot was taught to approach a drawer, grab the drawer, open the drawer, and then close the drawer.

During human–robot collaborations, a robot collects data and uses them to make decisions. Due to the non-deterministic nature of these data, machine learning is used for this processing [16]. The articles in this Special Issue demonstrate the power of machine learning to optimize task scheduling, detect collisions, collaborate with more than one person, and read social cues of a person. Scheduling tasks for human–robot collaborations in a production setting can be difficult as there are uncertainties that cannot be predicted and coded a priori offline, e.g., skill differences between human operators. Pupa’s online framework, which leverages the parallelism of human–robot collaboration, is one way to address this issue [10]. This novel framework has been shown to adapt to different human operator skills and reallocate task steps if the robot becomes unavailable. To accomplish this, a database was created to store the steps needed to accomplish a task. Then, a scheduler algorithm chose the most suitable task for each actor (robot or human), accounting for the operator’s skill level. The task monitoring component of the framework was fed back to the database to determine which details of the task were left to accomplish. While collaborating on these tasks, there are many points on articulated robots that can collide with the operator and cause injury. The location and magnitude of these collisions can be difficult to categorize. A neural network model has been previously developed to determine when a collision has occurred [17] so the robot can adjust its force and avoid an accident. Kwon expanded this neural network to include where on the robot the collision occurred [11]. This work is important for safety, especially as robots become more complicated with more articulations. Typically, these robots collaborate with a single human. Zou used N-player game theory to extend the collaborative ability of a robot to interact with two humans [12]. This theory utilized a recursive least-squares algorithm underlying a novel controller that allowed the robot to adapt to a human’s response. This controller was validated in a simulation where a robot helped two humans carry a table. Compared to a traditional linear quadratic regulator, this N-player game theory controller resulted in the humans exerting less effort. This work has the potential to extend beyond industrial robots to robots that help in homes. Akalin developed a reinforcement learning method to train robots that interact socially at home [13]. The robots were observed interacting with humans using a trial-and-error method to determine an optimal behavior. The robot learned which robot behaviors were desired through human feedback (e.g., facial expressions, vocal laughter) and stored this information in a database for later use. 

In summary, human–robot collaborations are a common occurrence. The articles in this Special Issue aim to increase the efficiency and safety of these collaborations. Sensors have been incorporated into the robots and surrounding workspaces so the robot can “see” the human. Humans have been outfitted with sensors as well, so they have additional data to “see” the robot. Finally, machine learning techniques have been developed so the robots can optimize these collaborations.

## Data Availability

Not applicable.
